# Children and adolescents’ preferences for support when living with a dying parent – An integrative review

**DOI:** 10.1002/nop2.1187

**Published:** 2022-02-13

**Authors:** Emily Beatrice Bergersen, Maria Larsson, Cecilia Olsson

**Affiliations:** ^1^ Department of Health Sciences Faculty of Health, Science and Technology Karlstad University Karlstad Sweden; ^2^ Section for Advanced Nursing Faculty of Social and Health Sciences Inland Norway University of Applied Sciences Elverum Norway; ^3^ Department of Bachelor Education Lovisenberg Diaconal University College Oslo Norway

**Keywords:** adolescents, children, palliative care, parenting, systematic review

## Abstract

**Aim:**

To identify and synthesize the evidence base regarding children and adolescents’ preferences for support when living with a dying parent.

**Design:**

Integrative literature review study.

**Methods:**

Searches were conducted in PubMed, CINAHL, PsycINFO, the Cochrane Library, Sociological Abstracts and Scopus, between 1 October 2019 and May 2021. Data were analysed and synthesized using integrative thematic analysis according to the analysis stages specified by Whittermore and Knafl.

**Results:**

Twenty‐two articles were identified. Children and adolescents’ preferences for support were described through one overarching theme, *Striving to achieve control and balance*, together with six subthemes; “Involvement in the sick parent's care and treatment”; “Wanting to be with the sick parent but needing respite”; “Information must be continuous and individually adapted”; “emotional and communicative support from parents and family members”; “professional, compassionate and informative support”; and “support in friendships and opportunities to maintain normality.”

## 1 INTRODUCTION

Living with a dying parent when one is at a young age is considered among the most traumatic experiences a person can have (Phillips, 2014). The loss can result in physical, cognitive, emotional and behavioural problems, and many children and adolescents, in this study defined as 2–18 years of age (Office of the High Commissioner for Human Rights, 1989; World Health Organization, 2001), struggle for years after the parent's death (Phillips, 2014; Torbic, 2011). This study was conducted from the children and adolescents’ perspective and focuses on their preferences for support during the period before and immediately after the parent's death. In this context, preferences are linked to children and adolescents’ first choice in how they want to be supported. This includes both support from professionals and other social networks. Such knowledge is needed when designing interventions to prevent ill health among bereaved children and adolescents and to improve the quality of palliative care. The consequences of not exploring different preferences may be that supportive interventions do not work as they should, and thus unsatisfactory help is generated for this vulnerable group.

## 2 BACKGROUND

Children and adolescents living with a parent who has a life‐threatening illness are at risk of developing long‐lasting symptoms and ill health. To eliminate outcomes such as low self‐esteem, behavioural difficulties (e.g., anger and aggression), long‐term illness or premature death caused by severe mental illness, substance abuse, self‐harm and suicide attempts, it is crucial to act preventively (Gupta, 2018; Jansson & Anderzén‐Carlsson, 2017; Simons et al., 2019). In addition, in a longer perspective, consequences are common regarding working life and economic situation as a result of difficulties to concentrate and learn in school, and low ambitions in relation to education and career planning (Brent et al., 2012). It is important to realize that children and adolescents represent the upcoming generation, and if their health is not taken seriously, this will lead to major international challenges in years to come (Høeg et al., 2019; Salam et al., 2016; Sawyer et al., 2012).

Modern treatment implies that patients in the palliative phase live longer than before, and many receive treatment at home. This also means that family members live in the shadow of illness, whether as informal carers or not, which can be experienced as a major burden. Despite this, few intervention studies consider the support of relatives (McFarlane & Liu, 2020; Northouse et al., 2010). Children and adolescents are a group of relatives who are particularly vulnerable. They undergo major physical, mental, social and cognitive changes as part of growing up, which must be balanced against living with a dying parent (EAPC, 2009; Etkind et al., 2017). They do not have the same abilities to deal with negative emotions as adults do, and are therefore at greater risk of developing complicated grief (Gupta, 2018; Salam et al., 2016).

As family members, children and adolescents can be considered a group that is often overlooked by healthcare professionals (HCPs) (Golsäter et al., 2019; Sawyer et al., 2012). Research describing children and adolescents’ situation and support needs from their perspective is scarce (Ellis et al., 2017; Kühne et al., 2012). Although there are guidelines for meeting this target group's needs and there is international consensus that HCP should be trained to promote (psychosocial) support to all members of the families of dying patients, it is apparently still difficult to deal with children and adolescents’ needs (Punziano et al., 2017; Valen et al., 2020). Accordingly, only a few studies have considered the need for measures to support children and adolescents before and after the death of the parent (Rosner et al., 2010; Spuij et al., 2015).

Furthermore, it has been pointed out that support for all family members should be extended throughout the grieving process and that HCP do not adequately support for children and adolescents as relatives of the dying (Bartfai Jansson & Anderzen‐Carlsson, 2017; Phillips & Lewis, 2015).

To summarize, that a parent's life‐threatening illness can negatively affect children and adolescents is supported by decades of research. Several studies have examined children and adolescents’ experiences of losing a parent, mostly reporting negative consequences for their health. In addition, there are a number of studies that focus grief and bereavement following the death of a parent (Brent et al., 2012; Jakobsen & Christiansen, 2011; Nickerson et al., 2013; Nilsson et al., 2009; Rostila et al., 2016). However, there is a need to summarize the evidence regarding children and adolescents’ preferences for support *when living* with a dying parent. In order to design supportive interventions that meet children and adolescents’ needs and have the potential to prevent negative consequences, synthesizing their preferences for support is necessary.

### 2.1 Aim

The aim of this study was to identify and synthesize the evidence base regarding children and adolescents’ preferences for support when living with a dying parent.

## 3 THE REVIEW

### 3.1 Design

An integrative review methodology was applied to explore the phenomena of interest from different perspectives using qualitative, quantitative and mixed‐methods studies (Whittemore & Knafl, 2005).

#### 3.1.1 Eligibility criteria

Inclusion criteria were selected based on the study aim. The included studies were conducted from the perspective of children and adolescents and focused on their preferences for support when living with a dying parent. The interventions were intended to provide physical, educational, emotional, cognitive, behavioural and social support. Only studies specifically mentioning children and adolescents in statements and quotations were included.

*Type of studies:* We included only primary research studies using qualitative, quantitative or mixed methods and written in English. Systematic reviews were excluded. There were no restrictions regarding the year of publication except the limits set by the databases (Table 1).

**TABLE 1:** Search method, databases and search terms

Search	Total number of records identified including duplicates	Total number of records identified excluding duplicates
**Databases:** PubMed, CINAHL, PsycInfo, Cochrane Library, Sociological Abstracts, Scopus. All searches up to 2021. **Search terms:** (“child” or “adolescent” or “minor” or “young person”) and (“bereavement” or “social support” or “adaption” or “psychological” or "Adaptation, Psychological" or “grief” or “experience(s)” or "Support, Psychosocial" or “Social Adjustment” or “Coping” ) and (“palliative care” or “hospice” or “palliative care nursing” or “hospice care” or “maternal death” or “parental death” or “death” or “terminal care”). **Limitations:** research article, title and abstract, age, language: English **Year limits set by databases:** PubMed; 1964–2021 CINAHL; 1973–2021 PsycInfo; 1800–2021 Cochrane Library; 1946–2021 Sociological Abstracts; 1922–2021 Scopus; 1970–2021	37,248	7,449

*Type of participants/settings:* Studies were included if their participants were 2–18 years old when they lived with a dying parent, had no known cognitive and/or mental illness, and had no secondary, non‐hereditary health problems before the parent became ill. They also had to be living in the same household as the parent. Studies focusing on mental disorders, substance abuse, suicide or sudden death were excluded. In addition, studies of parents with human immunodeficiency virus/acquired immune deficiency syndrome (HIV/AIDS) were excluded as, in these studies, children and adolescents often served as informal caregivers. Studies conducted in a non‐Western cultural context were likewise excluded.

#### 3.1.2 Search outcomes

The outcome was children and adolescents’ preferences for support and their experiences of existing interventions. Studies reporting parental death or expected death after a long period of incurable physical illness were included. Interventions and treatments managed and delivered by the municipal sector, hospitals, specialized palliative care units and patient associations to sick patients living at home were included.

### 3.2 Method

#### 3.2.1 Identification of relevant literature

##### Search methods

Literature searches were conducted between 1 October 2019 and 1 June 2020. A search update was performed in May 2021.

The literature was searched in PubMed, CINAHL, PsycINFO, the Cochrane Library, Sociological Abstracts and Scopus. These databases were selected as they cover multidisciplinary health care. Both MeSH terms (where available) and free text words were used. A specialist librarian was consulted when developing the search strategy to ensure rigour in the search process. References were handled using EndNote X8 software and Rayyan (Ouzzani et al., 2016). The search method is presented in Table 1.

##### Selection of literature

**FIGURE 1:**
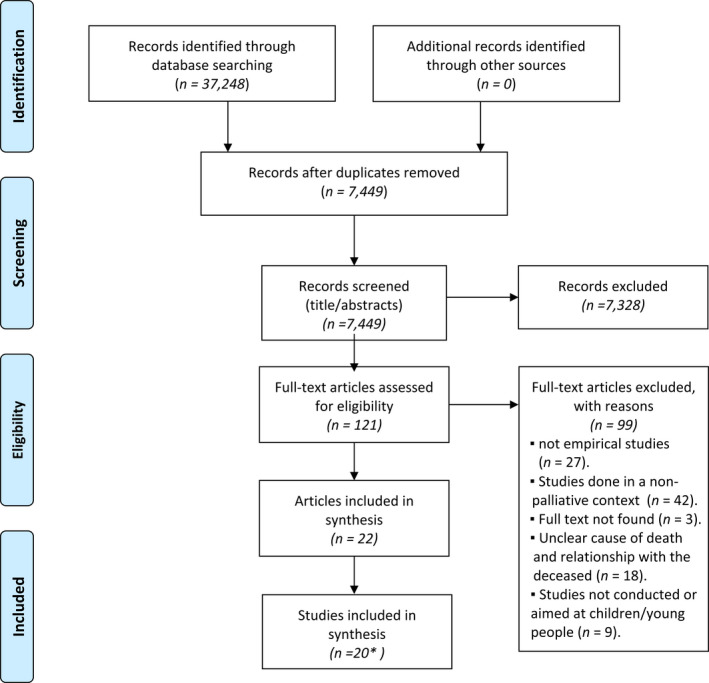
Flow diagram of the selection process. Source: modified from the flow diagram presented by Moher et al. (2009). *Two pairs of articles report research conducted on the same participants, so that the total number of research studies was 20

The flow chart in Figure 1 illustrates the selection of literature (Moher et al., 2009). The electronic database searches identified 7,449 articles, after removing duplicates. The authors screened titles and abstracts separately to ensure the inclusion of studies that met the study aim and inclusion criteria. Disagreement about inclusion was resolved by discussion until consensus was reached. Of the 7,449 articles, 7,328 did not meet the eligibility criteria and were rejected, leaving 121 articles for further reading. After assessing the 121 articles in full text, we ended up with 22 articles that met the study aim, four quantitative, 17 qualitative and one mixed‐methods.

##### Appraisal and data extraction

We used the Critical Appraisal Skills Programme (CASP) as modified by Nordström et al. (Nordström, Wilde‐Larsson, Sandsdalen, & Jansson, 2016a, 2016b) to assess the qualitative and quantitative studies. The CASP checklist tool consists of a simple scoring system that can contribute to a more objective quality assessment. Articles were rated as of high, medium or low quality. The findings’ validity and reliability were strengthened by having the three authors (EB, ML & CO) work closely together to ensure rigour in all stages of the review process. All steps of the selection process and of the appraisal and data extraction were performed independently (Higgins & Green, 2008; Whittemore & Knafl, 2005). Our review was registered in the PROSPERO International prospective register of systematic reviews (Bergersen et al., 2020).

##### Synthesis methods

The findings were synthesized and analysed according to the analysis stages specified by Whittemore and Knafl (2005), which allow data to be grouped by findings addressing the same phenomenon, rather than by method. This descriptive thematic analysis consists of data reduction, data display, data comparison, conclusion and verification.

The first step meant that data were organized into a manageable framework (i.e., data reduction). We reviewed the 22 articles in full text, using a self‐made form based on our research questions and inclusion criteria. The form assisted in classifying studies with diverse methodologies and enabled us to extract text from the articles in direct connection with our research questions. The form included a description of the method and sample as well as text (i.e., paragraphs and quotations) that answered our research questions.

Based on the findings from the reduction process, the data were summarized in a matrix (Table 2). The summaries answered all research questions in coherent text and served as a starting point for interpretation (i.e., data display).

**TABLE 2:** Summary of included articles

	Author and publication year	Country	Aim	Children and adolescents characteristics	Setting	Design & Method	Summary of relevant findings	Quality assessment
1.	Berman et al. (1988).	Canada	To describe the experiences of adolescents whose parents died of cancer.	Adolescents five male, five female – aged 11–17 (mean 14, 1) years.	Cancer clinic or palliative care unit	Qualitative semi‐structured questionnaire.	Adolescents want to be involved in their own terms. They seek support from families and other relatives, but HCPs seem to be distanced from them. Communication is an important factor. The time immediately after the parent had passed away was perceived as very demanding for many adolescents, at the same time as it also seems to lead to more freedom.	Medium
2.	Christ et al. (1994).	USA	A summary of the characteristic psychosocial reactions to a parent's advanced illness (as part of a research program on childhood bereavement, evaluating the impact of a parent guidance intervention design to facilitate adjustment to parental death).	One hundred and twenty adolescents aged 11–17 years from 86 families.	Cancer centre	Qualitative individual interviews.	Adolescents want to be independent, but feel guilty because of this. They want to distinguish between normality and disease. Some seek information and understanding, and gain support from both the family and professionals, while others do not want to be involved at all. The terminal stage of the illness is the most challenging period.	Low
3.	Semmens and Peric (1995).	Australia	To gain insight into, and understanding of, children's perspective of a parent's chronic illness and subsequent death.	A convenience sample of four females and one male aged 13–20 years.	Home‐setting	Qualitative individual interviews analysed using Colaizzi's seven‐step phenomenological methodology.	There is a lack of knowledge among adolescents and they want honest and age‐appropriate information. Spending time with the ill parent and being able to say goodbye are important. Following the parent's disease development closely makes them better prepared for changes. Making happy memories and accepting the individual grief process are highlighted.	High
4.	Saldinger et al. (2003).	USA	To find out how school‐aged children manage the strain of exposure to traumatic stimuli during the anticipated death of a parent.	Surviving parents and their children (limited to two children per family). Family members were interviewed 8–36 months after the death when the children were aged 6–16 years (a total sample of 58 parentally bereaved school‐aged children).	Home‐setting	Qualitative semi‐structured interviews collected as part of the Wave One and Wave One Extended of the Family Style Project (intervention).	There is great variety in children and adolescents’ psychological responses to stimuli such as watching a parent die. They can feel guilty about not wanting to visit their parent, and struggle to balance reality and imagination. Children should not be pushed and their needs must come first. However, this is not easy when you live with the sick parent, and many want to escape from the situation from time to time.	Medium
5.	Beale et al. (2004).	USA	To describe the experience of 28 children/siblings of parents/siblings with terminal cancer and child bereavement following the loss of a parent.	Twenty‐eight children and adolescents aged 3–18 years who experienced the impact of having a parent or sibling with terminal cancer.	Unknown	Qualitative individual interviews.	The availability of family and professionals is important. Children and adolescents need help to cope with their emotions and want honest information. Some also find it positive to be a caregiver for their sick parent. Children and adolescents need support early in the illness rather than in the terminal phase (when there are too many negative emotions).	Medium
6.	Bugge et al. (2008).	Norway	To assess a preventive support programme for children aged 5–18 years and their families when a mother or father has an incurable form of cancer.	Twelve children and adolescents, eight girls and four boys, aged 6–16 (mean 9) years. For five of the children, their mother, and for seven, their father was ill.	Hospital or home setting	Qualitative in‐depth interviews after participation in the Family Support Programme (intervention).	Children and adolescents want to know the prognosis, and what their own reactions might be. They want to live as normally as possible despite the illness. They prefer support from people outside their family so they can talk honestly and openly. Children and adolescents realized that their parents were preoccupied, and they wanted to protect them by not sharing all their thoughts and feelings.	High
7.	Dehlin and Reg (2009).	Sweden	To describe adolescents’ experiences of the serious illness and death of a parent.	Five adolescents, two girls and three boys, who were 14–17 years old when one of their parents died. At the time of the study, they were 16–18 years old.	The parent had been hospitalized in western Sweden.	Qualitative interviews.	Having the sick parent at home is a good thing as it gave them a chance to make happy memories. The adolescents wanted friends to show more interest, but at the same time they needed privacy and did not want to be pitied. They felt that the support system was mainly for their parents, but appreciated talking to older relatives who had a relationship with the sick parent (sharing memories). Support throughout the sickness period is recommended.	High
8.	Popplestone‐Helm and Helm (2009).	England	To reflect on the benefits and challenges of setting up and running a support group, and on the journey of the hospice (St. Richard's Hospice) to date.	Children and adolescents, who have participated in the ‘Inside‐Out Group’ (unclear description of the number of participants).	Hospice	Quantitative questionnaire as feedback on a support group provided by St. Richard's Hospice (intervention).	The intervention helped the children and adolescents make new friends and think better thoughts about the sick parent. It was helpful talking to peers who were coping with the same feelings, but at the same time it was difficult to talk about and listen to others’ sad emotions. The intervention could help ease the pain.	Low
9.	Patterson and Rangganadhan (2010).	Australia	To identify and better understand the needs of adolescents and young adults who have lost a parent to cancer, and to assess the extent to which these needs had been met in the study population.	Sixty‐two parentally bereaved adolescents aged 12–23 years. All participants were recruited from Canteen, an organization for young people living with cancer.	Home‐setting	Qualitative questionnaire ( open‐ended).	Adolescents want more support and understanding from others. They want help coping with emotions and to talk to people who understand them such as a peer. They want more information about the disease, and to be able to have “time out” and time to grieve on their own terms. Help with increased household responsibilities was also mentioned.	High
10.	Keeley and Baldwin (2012).	USA	To examine messages of everyday communication (small talk and routine interactions).	Forty‐one female and 20 male children and adolescents, aged 6–18 years, who had lost a mother (14%), father (43%), sibling (24%) or extended family member (19%) after an extended period of illness. Interviews took place 2 months to 3 years after loved one's death.	Hospice	Qualitative retrospective interviews.	Everyday communication gives children and adolescents security and comfort. It is an avenue for openness and normalcy. Everyday communication leading up to the parent's death may become a family ritual. It can create happy memories and the participants missed it after their loved one's death.	Medium
11.	Buchwald et al. (2012).	Denmark	To describe and understand how children handle their life when a mother or father is dying.	Seven children and adolescents, four boys and three girls, aged 11–17 years and living with a seriously ill and dying parent.	Hospice or home setting	Qualitative interviews and video diaries.	Children and adolescents want to spend time at home with the sick parent, but at the same time they need to be able to leave the house briefly to forget their fear. Some tried to keep distance from the disease, while others tried to influence the situation. The worst period was when the parent was diagnosed and when the illness took a turn for the worse.	High
12.	Karlsson et al. (2013).	Sweden	To describe young adults’ perspectives on the experience of having a parent who developed cancer when they (i.e., the young adults) were adolescents.	Six adults, one man and five women, aged 20–26 years. The inclusion criterion was experience, during adolescence (age 13–19 years), of a parent being diagnosed with cancer.	Home‐setting	Qualitative narrative interviews.	Adolescents want to learn about the disease, preferably from professionals. They want to share memories of the parent with their friends and talk to someone who understands. Being involved in the treatment is desired, but at the same time they need to retain some normalcy. They do not want to talk to someone they do not know, and the parents, both the healthy and the sick ones were often the children and adolescents’ first choice as support persons. After the death, they gain a new normalcy.	High
13.	Melcher et al. (2015).	Sweden	To describe how teenagers are emotionally affected by, and how they try to adapt to, everyday life in a family where a parent is dying.	Ten adolescents (14–19 years old, seven boys and three girls), four of whom had lost their mother and three their father. Three of the deceased were not biological parents, but had shared in household and family life.	The patients all had advanced cancer and received home care as well as intermittent inpatient care (at the palliative care unit)	Qualitative interviews.	Talking to the dying parent helped the adolescents prepare for the loss. Taking on responsibilities in the home led to personal growth. They tried to arrange for the parent to be cared for at home. Support from the parents was the most important, but also from others in and around the family (not professionals). The information given must be honest and consistent.	High
14.	Kopchak Sheehan et al. (2014).	USA	To describe four ways in which parents disclose information about one parent's life‐threatening illness to their adolescent children.	Sixty‐one hospice patients, their spouses, and their adolescent children. Adults in the hospice programme were eligible to participate if they had children aged 12–18 years; could speak, write, and understand English; and had the cognitive ability and physical stamina to complete the interview. Parent surrogates, including grandparents or significant others in a parenting role, were included in the study.	Hospice	Qualitative interviews.	Some adolescents were gratified that their parents shared important information with them; others did not want to discuss the illness at all. Some preferred practical to emotional information. The worst approach was inconsistent information.	Medium
15.	Bylund‐Grenklo et al. (2015).	Sweden	To investigate cancer‐bereaved youths’ opinions about and experiences of being told about a parent's imminent death from cancer, and of barriers to this communication.	A total of 622 adolescents who at age 13–16 had lost a parent to cancer (6–9 years previously). Participants had to: have lived with both parents at the time of the loss; have one living parent at the time of the survey; and have an identifiable telephone number.	Register	Quantitative Nationwide population‐based survey.	Adolescents have a great desire for information. This includes the desire to be prepared, avoid negative surprises, have a chance to talk and say farewell, and improve understanding and coping. End‐of‐life medical information to the family, preferably both before and after the loss, is associated with higher trust in the health care provided, which in turn is associated with a lower risk of depression.	High
16.	Sheehan et al. (2016).	USA	To generate an explanatory model of the coping strategies that adolescents employ to manage the stressors they experience in the final months of their ill parent's life and shortly after the parent's death.	Altogether 26 families of adolescents with a parent receiving care in a large hospice programme: 14 ill parents, 17 well parents/guardians and 30 of their adolescent children before the parent's death; additionally, six of these families after the death.	Hospice	Qualitative semi‐structured interviews.	Some adolescents tried to stay busy and live as normally as possible, while some wanted to care for the dying parent. The adolescents’ needs changed throughout the illness trajectory. Their need for support increased with deterioration of the parent's illness.	High
17.	Shallcross et al. (2016).	USA	To conduct a multi‐site, quantitative evaluation of the effects of the CLIMB intervention on parent/caregiver reports of children's emotional symptoms and conduct problems, and children's reports of four domains of emotion regulation, using a pre–post design. Secondary outcomes included a quantitative and qualitative evaluation of parent and child satisfaction with the CLIMB intervention.	Forty‐five children aged 6–11 who had a primary caregiver with cancer (grandparent, uncle, aunt, unrelated adult).	Medical Centre	Quantitative survey and seven questionnaires/scales to measure the effects of the CLIMB programme on behavioural functioning and emotion regulation.	The intervention led to some improvement in emotion regulation. Feedback from caregivers and children reflected overall satisfaction. Children's qualitative responses indicated that they appreciated the art and crafts component of the programme, the social support and the emphasis on discussing feelings.	High
18.	Sveen et al. (2016).	Sweden	To explore how teenagers reason about a parent's recent death and about their life without that parent.	Ten adolescents (seven boys and three girls aged 14–19 years) were interviewed twice, 3–12 months after their parent's death.	The parents all had advanced cancer and received home care as well as intermittent inpatient palliative care	Qualitative interviews.	Some wanted to remember the parent as healthy and did not want to talk about the disease. Others talked to family and counsellors/therapists. HCPs did not approach them. For some, the worst period was the time of the death; for others, it was the period immediately afterwards. Death had ended the parent's suffering and life with severe illness. The parent's death was also a relief for the adolescents, as they could let go of all the strain and return to everyday life.	High
19.	Tillquist et al. (2016).	Sweden	To describe female teenagers’ experiences of losing a parent to cancer.	Five blogs written by girls aged 13–19 years who had lost a parent to cancer.	Blogs	Qualitative content‐analysis.	Adolescents want to spend time with their ill parent and have a great desire for information. The importance of saying goodbye and getting support from friends and professionals was highlighted. Honouring the memory of the parent gave motivation. The worst part was when the HCPs did not manage to eliminate or relieve the pain the parent was experiencing. After death, they described feelings of guilt, shame and selfish behaviour due to a lack of strength.	Medium
20.	Alvariza et al. (2017).	Sweden	To explore young adults’ advice to HCPs on how to support teenagers who are losing a parent to cancer.	Altogether, 481 adolescents who had lost a parent to cancer when they were 13–16 years old. The participants were aged 18–26 years at the time of the study, and they had lost their parents 6–9 years previously.	Register	Qualitative descriptive/interpretive design. This work was based on a nationwide survey with open‐ended questions.	HCPs should take the first step to communicate with and see each adolescent individually. They should have knowledge and understanding of adolescents’ feelings and reactions, not feel sorry for them, and occasionally approach them with unrelated small talk. Adolescents want honest and understandable information and to know as much as possible about the parent's illness. The HCPs should invite adolescents to family meetings or even have individual conversations and provide time with the ill parent. Adolescents should be offered individual psychosocial support. Also, HCPs should take time to talk with them and provide information continuously throughout the illness trajectory and inform them when their parent's condition deteriorates.	High
21.	Eklund, Jalmsell, et al. (2020).	Sweden	To explore children's reports of illness‐related information and family communication when living with a parent with a life‐threatening illness.	Forty‐eight children and adolescents, aged 7–19 years.	Palliative home care Units	Baseline survey data from an ongoing intervention study.	Children and adolescents wanted more insight into their parent's illness. They reported not been given enough information, and many of the Children and adolescents reported having no family member with whom they could express or talk about their own feelings. To support these Children and adolescents, and to encourage parents to talk to their children and adolescents, family‐focused interventions to promote family communication and improve communication between family members and HCP are needed, preferably shortly after the parent's diagnosis or early in the illness trajectory and offered by skilled and courageous clinicians.	High
22.	Eklund, Kreicbergs, et al. (2020).	Sweden	To explore the feasibility of the family talk intervention (FTI) and its acceptability to dependent children when a parent is cared for in palliative home care.	25 children and adolescents, aged 6–19 years.	Specialized palliative home care units	A convergent mixed‐method design was used involving both questionnaires and interview data.	A majority of the children and adolescents appreciated the structure and content of FTI. The intervention came at the right time, consisted of the right amount of meeting points and met the expectations they had to a large extent. They felt seen, heard, and acknowledged by the interventionists. They also appreciated talking about things that were not necessarily related to the parent's illness, such as school, leisure activities, and the like. They felt it was good to talk to someone about the feelings they had and recommended FTI to other children and adolescents in similar situations.	High

Abbreviations: CLIMB, Children's Lives Include Moments of Bravery, a programme to support children of parents with cancer; HCP, healthcare professional.

The next step was to identify patterns, themes and relationships based on the data display. Through several rounds of reviewing the data, we ended up with 80 statements coded in relation to the research aim and questions. These codes were compared to reveal similarities and differences, and were then grouped into themes and patterns. Tables were created for each round of analysis to make it easier to follow the process and the development of themes (i.e., data comparison).

**FIGURE 2:**
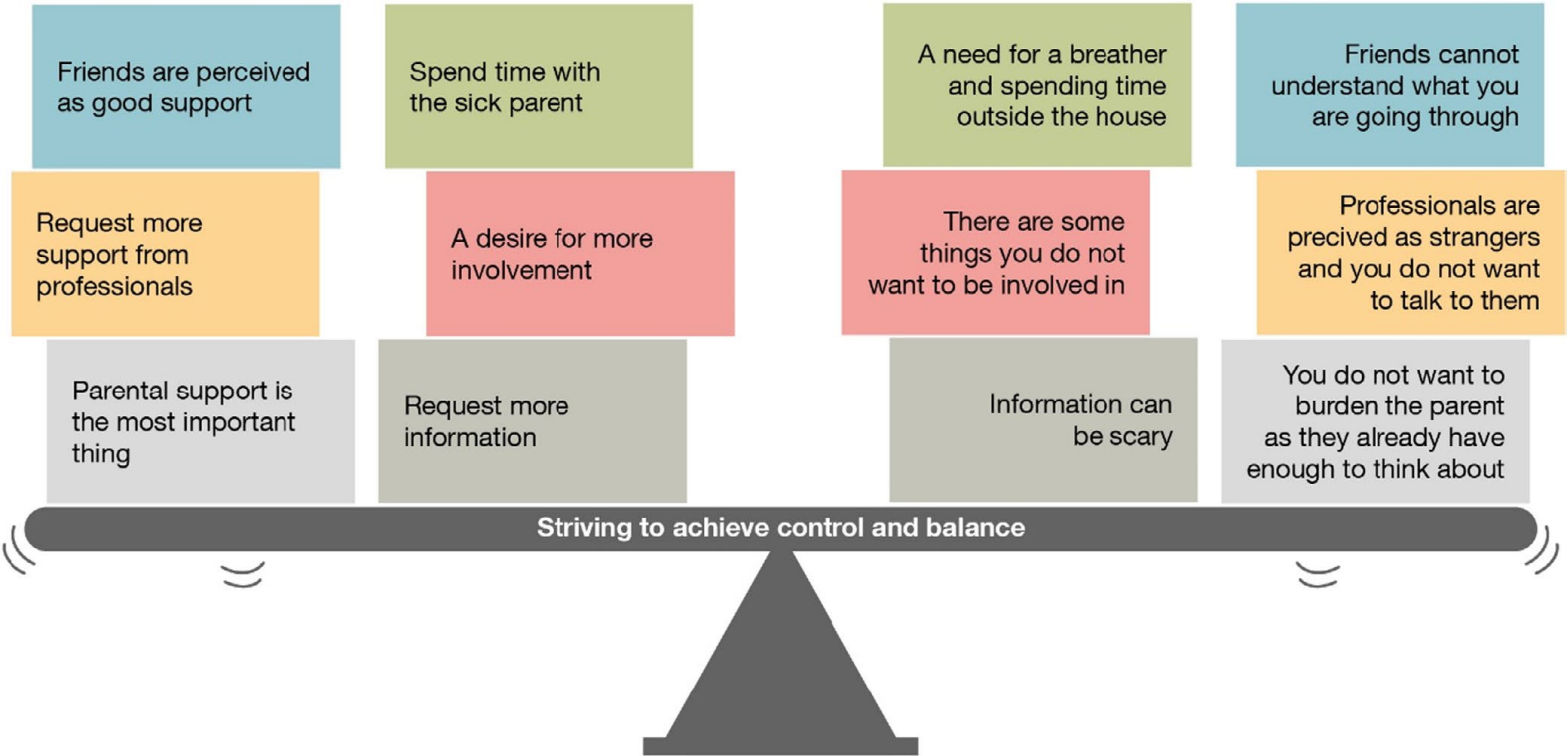
Illustration of contradictions in children and adolescents’ preferences for support

Finally, themes and subthemes were identified and their internal relationships were described using a higher level of abstraction, moving from the particular to the general. In this step also variations within the subthemes were identified, i.e., contradictions in children and adolescents’ preferences for support. In order to illustrate and enable understanding of the results, a graphic model was developed (Figure 2). The authors (EB, ML & CO) did this jointly, after separately assessing the themes (i.e., conclusion and verification).

## 4 RESULTS

### 4.1 Characteristics of the included articles and studies

The included articles (*N* = 22) were published between 1988 and 2020 (Tables 2 and 3). The quality of the selected articles was low (*N* = 2), medium (*N* = 6) and high (*N* = 14). Seventeen (77%) articles used qualitative research designs, four (*N* = 18%) used quantitative designs and one used mixed‐methods (*N =* 5%). Two pairs of articles, i.e., (1) Melcher et al. (2015) and Sveen et al. (2016) and (2) Bylund‐Grenklo et al. (2015) and Alvariza et al. (2017), report research conducted on the same participants, so the total number of research studies was 20. The following presentation of percentages is calculated based on the number of studies (*N* = 20).

**TABLE 3:** Characteristics of children and adolescents in included articles.

	Demographic data	Articles reported (*N* = 22)	Results of articles reported
Articles	Geographical data	22	Canada (*N* = 1) USA (*N* = 7) Australia (*N* = 2) Norway (*N* = 1) Sweden (*N* = 9) England (*N* = 1) Denmark (*N* = 1)
Children and adolescents	Age	22	3–26 years
	Gender	18	46.4% Male 53.6% Female
	Parent's diagnosis	15	Cancer (*N* = 12). Other diagnoses^a^ (*N* = 3)
Setting	Rural or urban	2	Urban (*N* = 2)
	Services	18^b^	Home setting (*N* = 8) Cancer clinics/palliative care unit/hospital (*N* = 14).

^a^ Alcoholism death, juvenile diabetes, degenerative arthritis, diverticulosis amyotrophic lateral sclerosis (ALS), multiple sclerosis (MS), pulmonary disease and bovine spongiform encephalopathy (BSE).

^b^ In four of the articles, more than one setting was described.

The studies were from Canada (*N* = 1, 5%), the USA (*N* = 7, 35%), Australia (*N* = 2, 10%), Norway (*N* = 1, 5%), Sweden (*N* = 7, 35%), the UK (*N* = 1, 5%) and Denmark (*N* = 1, 5%). The studies were evenly distributed over the reviewed time range, but most of them were published from 2000 onwards (86%). Only two studies (10%) specified whether the research had been conducted in an urban or rural setting (urban *N* = 2).

It was possible to calculate a total of 1,159 participants in the studies in aggregate. In Kopchak Sheehan et al. (2014) and Popplestone‐Helm and Helm (2009), the number of participants was not clear. Participants were 3–26 years old when data were collected but below 18 years when their parent was dying.

The diagnosis of the dying or diseased parent is specified in 15 of the 20 studies. Most of the diagnoses were cancer. Other life‐threatening diseases reported were amyotrophic lateral sclerosis (ALS), multiple sclerosis (MS), pulmonary disease, bovine spongiform encephalopathy (BSE), alcoholism, juvenile diabetes, degenerative arthritis and diverticulosis.

All participants lived with the sick parent throughout the illness. The parent received intermittent treatment at various institutions such as cancer clinics or palliative care units, and many participants were recruited through these. Data were also collected from official registers and blogs. “Home setting” in Tables 2 and 3 indicates that the interviews were conducted at home or that the participants themselves had applied for inclusion in the study.

A total of five intervention studies were included (Bugge et al., 2008; Eklund, et al., 2020; Popplestone‐Helm & Helm, 2009; Saldinger et al., 2003; Shallcross et al., 2016), in four of which the intervention was clearly described. All these studies had a family focus with children and adolescents included among the participants. The interventions were based on theories concerning traumatic stress, psycho‐education, pre‐death support, psychological/behavioural functioning and emotion regulation. The participants seemed satisfied with their participation in the interventions. Cost‐effectiveness was not emphasized in any of these studies.

### 4.2 Thematic findings

Children and adolescents’ preferences for support were described through one overarching theme, *Striving to achieve control and balance*, together with six subthemes; “Involvement in the sick parent's care and treatment”; “Wanting to be with the sick parent but needing respite”; “Information must be continuous and individually adapted”; “emotional and communicative support from parents and family members”; “professional, compassionate and informative support”; and “support in friendships and opportunities to maintain normality.” The subthemes were raised in various ways by different children and adolescents and there were contradictions in preferences for support and ways to strive expressed both within and between different children and adolescents. Figure 2 describes the thematic findings, presenting a selection of quotations that illustrate these contradictions.

#### 4.2.1 Striving to achieve control and balance

Control was described as allowing children and adolescents to choose for themselves how involved they wanted to be during their parents’ course of illness. This related to information, experiences such as watching the parent die, and practical involvement, such as spending time with the sick parent and having responsibilities around the house. Some mastered the situation through considerable involvement, while others felt better off not being too involved. In addition, control concerned maintaining everyday life as much as possible, by leaving the house, going to school and spending time with friends. This can be seen as a way to keep a form of normality and familiarity in an otherwise chaotic existence: children and adolescents who feel that they have control may find it easier to balance their own life against a parent's illness.

The time point varied as to when children and adolescents were in greatest need of support. Some found the worst period to be when the parent was diagnosed (Beale et al., 2004; Buchwald et al., 2012; Eklund, et al., 2020; Tillquist et al., 2016), others when the illness took a turn for the worse, especially when there were major changes (i.e., tipping points) (Sheehan et al., 2016; Tillquist et al., 2016). The terminal stage and actual death was the worst for some, while the time immediately after the death was the worst for others (Berman et al., 1988; Christ et al., 1994; Karlsson et al., 2013; Sveen et al., 2016). Support throughout the parent's sickness period is recommended (Alvariza et al., 2017; Dehlin & Reg, 2009).

##### Involvement in the sick parent's care and treatment

This subtheme captured varied needs regarding how children and adolescents want to be involved in care, treatment and/or funeral planning, and also captured the extent to which children and adolescents could handle watching physical and mental changes occur in the sick parent.

Some children and adolescents preferred practical involvement, such as helping with the treatment or organizing the funeral, rather than emotional involvement (Kopchak Sheehan et al., 2014), while others wanted to stay separate from the illness and did not want to be involved at all (Christ et al., 1994; Dehlin & Reg, 2009; Kopchak Sheehan et al., 2014; Sveen et al., 2016). Considerations about what a child should and should not be exposed to are important, they should not be pushed and their needs must come first. This could be linked to stimuli such as seeing the parent become very ill, witnessing frightening treatments, or upsetting aspects of the hospital environment (Berman et al., 1988; Christ et al., 1994; Saldinger et al., 2003). Several children and adolescents were given the role of caregiver and said that it was challenging to see the parent in pain (Saldinger et al., 2003). Some wanted to remember the parent as healthy (Sveen et al., 2016). There is no way to handle exposure to a dying parent that will fully protect a child, and children and adolescents often struggle to balance reality and imagination (Saldinger et al., 2003). They can jump to their own conclusions without getting confirmation, and experience this as frightening (Dehlin & Reg, 2009).

##### Wanting to be with the sick parent but needing respite

This subtheme showed that children and adolescents differ in preferences regarding spending time with the sick parent and the need for time alone outside the home. Most seemed to prefer living with the sick parent at home. By being close to the parent, they could follow the illness trajectory and be prepared for changes (Semmens & Peric, 1995) and were able to know immediately when something had changed, which led to a feeling of security (Buchwald et al., 2012) and being a caregiver helped them deal with anxiety and grief (Beale et al., 2004; Sheehan et al., 2016). Spending time with the sick parent gave them a chance to make happy memories (Alvariza et al., 2017; Bylund‐Grenklo et al., 2015; Dehlin & Reg, 2009) and talk about important things (Alvariza et al., 2017). Some did not want the parent to die in hospital (Melcher et al., 2015), both to honour the parent's wishes and because the hospital felt intimidating to them (Melcher et al., 2015; Saldinger et al., 2003). Some mentioned the importance of being able to say goodbye (Alvariza et al., 2017; Bylund‐Grenklo et al., 2015; Semmens & Peric, 1995).

The children and adolescents strived for a sense of normality, without constant present thoughts of severe disease and death, which was challenging as they lived with their dying parent (Christ et al., 1994; Sheehan et al., 2016). There was also a lot of guilt associated with living close to the sick parent, as some children and adolescents experienced a desire to escape from home and the situation in general (Buchwald et al., 2012; Christ et al., 1994; Saldinger et al., 2003). Children and adolescents are constantly evolving, and resuming activities and developmental tasks is important (Berman et al., 1988; Buchwald et al., 2012; Bugge et al., 2008; Christ et al., 1994; Dehlin & Reg, 2009; Patterson & Rangganadhan, 2010; Sveen et al., 2016). The children and adolescents needed to have fun and spend time with friends (Berman et al., 1988; Buchwald et al., 2012; Christ et al., 1994; Dehlin & Reg, 2009; Patterson & Rangganadhan, 2010; Sveen et al., 2016).

The situation at home could also lead to conflicts within the family (Christ et al., 1994), even though the family members were in a situation that required mutual closeness and support. With the sick parent living at home, the household chores often increased and this further limited the children and adolescents’ independence. Some children and adolescents said they wanted help with household chores (Berman et al., 1988; Christ et al., 1994; Dehlin & Reg, 2009; Melcher et al., 2015; Patterson & Rangganadhan, 2010), while others appreciated being able to contribute with household chores as doing so gave them a sense of responsibility and maturity (Berman et al., 1988; Christ et al., 1994; Melcher et al., 2015; Patterson & Rangganadhan, 2010; Semmens & Peric, 1995).

##### Information must be continuous and individually adapted

This subtheme showed that information must be tailored to the individual children and adolescents needs and to the details of the parent's illness, diagnosis, prognosis and (imminent) death (Alvariza et al., 2017; Berman et al., 1988; Eklund, Kreicbergs, et al., 2020; Patterson & Rangganadhan, 2010; Semmens & Peric, 1995; Tillquist et al., 2016). The information must enable the individual children and adolescents to understand what is happening and be prepared for what is to come. Not knowing and not being understood can lead to strong emotions such as loneliness, anger and frustration (Karlsson et al., 2013; Semmens & Peric, 1995). The information must be honest and consistent (Beale et al., 2004; Bugge et al., 2008; Karlsson et al., 2013; Kopchak Sheehan et al., 2014; Melcher et al., 2015; Semmens & Peric, 1995). The information must also be age‐appropriate (Semmens & Peric, 1995) and be given on the children and adolescents’ own terms (Dehlin & Reg, 2009). Although the need for information is generally great among children and adolescents, some prefer not to know everything while some said that they had received information they did not want to know (Dehlin & Reg, 2009; Eklund, Jalmsell, et al., 2020).

##### Emotional and communicative support from parents and family members

This subtheme captures the contradiction between wanting support from parents and not wanting to burden them in an already challenging life situation.

The parents, both the healthy and the sick ones, were often the children and adolescents’ first choice as support persons (Berman et al., 1988; Christ et al., 1994; Eklund, Kreicbergs, et al., 2020; Karlsson et al., 2013; Keeley & Baldwin, 2012; Melcher et al., 2015; Semmens & Peric, 1995). However, the children and adolescents realized that their parents were preoccupied, and they wanted to protect them by not sharing all their thoughts and feelings (Bugge et al., 2008). Some also experienced that they could only talk a little, or not at all, with family members (Eklund, Kreicbergs, et al., 2020). They had support from family, friends and acquaintances (Berman et al., 1988; Karlsson et al., 2013; Melcher et al., 2015; Tillquist et al., 2016), but meeting someone who could truly understand, such as a peer, was preferred (Berman et al., 1988; Karlsson et al., 2013; Patterson & Rangganadhan, 2010; Popplestone‐Helm & Helm, 2009; Shallcross et al., 2016). Support persons also included other family members with whom the children and adolescents could share memories (Dehlin & Reg, 2009). It could also be challenging to be honest with family members about their thoughts and feelings. Some wanted more support from their parents, finding that their parents were unable to answer all their questions (Eklund, Jalmsell, et al., 2020; Eklund, Kreicbergs, et al., 2020; Karlsson et al., 2013; Patterson & Rangganadhan, 2010).

##### Professional, compassionate and informative support

This subtheme describes a desire for more support from HCPs, who were simultaneously perceived as strangers not comfortable sharing private things.

There seemed to be a perception among children and adolescents that the support system was mainly for their parents (Dehlin & Reg, 2009; Sveen et al., 2016) and that HCPs did not play an important role for them (Melcher et al., 2015). The children and adolescents believed that HCPs had done all they could for the sick parent, but found it difficult when they could not eliminate or relieve the pain experienced by the parent (Sveen et al., 2016; Tillquist et al., 2016). Most children and adolescents seemed to have support from counsellors/psychotherapists and school staff (Beale et al., 2004; Bugge et al., 2008; Christ et al., 1994; Sveen et al., 2016; Tillquist et al., 2016). However, several participants said they wanted more support from HCPs. They wanted HCPs to demonstrate concern (Alvariza et al., 2017; Berman et al., 1988; Eklund, Jalmsell, et al., 2020) and to be honest, provide information, and be aware of individual needs (Alvariza et al., 2017; Berman et al., 1988; Bylund‐Grenklo et al., 2015; Eklund, Kreicbergs, et al., 2020; Karlsson et al., 2013). However, they did not want to be pressured to talk and/or answer demanding questions (Berman et al., 1988); instead, they said that HCPs should occasionally approach them with small talk, not always focusing on the illness (Alvariza et al., 2017). Some children and adolescents found it frightening to talk to a stranger, such as a HCP, especially at the beginning of the illness course (Bugge et al., 2008; Karlsson et al., 2013). Several studies emphasized that information should be provided by HCPs (Alvariza et al., 2017; Berman et al., 1988; Bylund‐Grenklo et al., 2015; Christ et al., 1994; Karlsson et al., 2013; Tillquist et al., 2016). However, children and adolescents stressed that they preferred talking to experienced HCP well‐trained in communication (Eklund, Jalmsell, et al., 2020).

Help addressing negative emotions was greatly needed (Alvariza et al., 2017; Beale et al., 2004; Bugge et al., 2008; Christ et al., 1994; Popplestone‐Helm & Helm, 2009; Saldinger et al., 2003; Shallcross et al., 2016). This was related to a desire not to feel different and to retain control over one's personality.

##### Support in friendships and opportunities to maintain normality

This subtheme illustrates how friends can be an important source of support, or may not understand what children and adolescents are going through when living with a parent who has a life‐threatening disease.

Support from friends was two‐folded: many found friends to be a great support (Berman et al., 1988; Karlsson et al., 2013; Tillquist et al., 2016); at the same time, friends might not fully understand what the children and adolescents were going through, so it was difficult to turn to them (Berman et al., 1988; Dehlin & Reg, 2009; Patterson & Rangganadhan, 2010). It was also important for the children and adolescents not to be treated differently from usual, including being pitied (Alvariza et al., 2017; Dehlin & Reg, 2009; Patterson & Rangganadhan, 2010; Sveen et al., 2016), but they also did not want to be forgotten or feel alone. Friends were seen as a “protected zone” because they generally did the same things as before and acted as usual. Being with them enabled the children and adolescents to stop thinking about their parents’ illness for a while (Dehlin & Reg, 2009). On the other hand, it was important that their friends should understand and cared, and that they listen to them. The children and adolescents sometimes experienced anger and sadness when their friends talked about their own parents (Patterson & Rangganadhan, 2010). Although many liked to talk to other people who were undergoing experiences similar to theirs, they did find it difficult to listen to others’ sadness (Popplestone‐Helm & Helm, 2009).

#### 4.2.2 Synthesis of findings

The synthesis of findings revealed various preferences for support among children and adolescents living with a parent who has a life‐threatening disease. Preferences can be influenced by self‐image, maturity, perceived security, coping strategies and social environment. However, a common finding was that the children and adolescents were in a complex and demanding situation and therefore strove for control and balance in their lives. *Striving to achieve* this *control and balance* is illustrated by the tilting plank in Figure 2, showing how ongoing striving took place in several areas (i.e., subthemes) simultaneously. This illustrates both the struggle within individuals and the variation between individuals, but it also illustrates what nurses and other HCP face when meeting the target group.

## 5 DISCUSSION

The aim of this study was to identify and synthesize the evidence base regarding children and adolescents’ preferences for support when living with a dying parent. Children and adolescents’ preferences for support were unique, as reported elsewhere (Warnick, 2015).

This integrative review shows that the most important source of support for the majority of children and adolescents seems to have been their parents. This can be expected, as children and adolescents are dependent on their parents’ care (Niemelä et al., 2010). Involvement of parents in interventions has also proved to be a decisive factor in bereavement care (Chen & Panebianco, 2018). This may partly explain why several included studies had both adult and young participants, and why interventions often target the family as a whole. Family‐oriented care is used and supported by many, and is relatively well recognized in palliative care (Benzein et al., 2008; Dumont & Kissane, 2009; Hudson et al., 2008). Results of this study indicate that it can be challenging for parents to balance caring for their children and being ill, which is also supported by previous research (Elmberger et al., 2008). It is also worth mentioning that the definition of the nuclear family is changing, and that the family perspective should also be extended to deal with, among other things, children and adolescents of divorced parents and the challenges they may entail (Marcussen et al., 2020). Our results indicate that it can be difficult for children and adolescents to be honest about their feelings and preferences when other family members are present. It is therefore worth considering whether a person‐centred or child‐centred approach may be better for meeting the preferences of children and adolescents for support. An important difference between these concepts, however, is that person‐centred care is aimed at adults with autonomy, which children under the age of 18 do not have. In a child‐centred care, one must still respect the child's individual rights, despite the fact that they are still dependent on their family (Coyne et al., 2018). Regardless of the form of “centeredness” as a procedure, the concept must be modified somewhat to bring out the relatives' perspective, especially when the relatives' is children and adolescents, rather than focusing solely on the sick patient.

Changes in family structure can be challenging and often result in new self‐understandings and new life‐worlds for everyone involved. They bring about a transition between what came before and what is evolving. The time between two stable phases – i.e., the end of the familiar and the beginning of something new – is often marked by an unstable period. The process is unique to each individual and, in the case of serious illness, is often complex. During the transition, there is a clear need for new knowledge, changed behaviour, a supportive environment and the re‐examination of one's self‐image (Kralik et al., 2006; Meleis, 2010). Although we did not find a clear indication as to when support should be given, we did discern some signs that the need is in conjunction with “tipping points” in the parent's illness trajectory, as this is when children and adolescents are most vulnerable. Nurses and other HCP should be aware of this when supporting children and adolescents. Gaining an overview of how transitions are experienced within the family can be useful (Werner‐Lin et al., 2010). It is also worth noting that transitions can be linked to normal life changes and personal development (Meleis et al., 2000). In childhood and adolescence, everyone goes through cognitive, physical and emotional changes, which in itself can be demanding. Children and young people living with a life‐threatening sick parent are thus forced to balance different transition processes.

This integrative review shows that support to children and young people living with a life‐threatening sick parent is comprehensive and few interventions were identified in the literature that shed light on this matter. In addition, the majority of the interventions identified were aimed at the family rather than the individual child or adolescent – which may make it difficult to reveal the individual's preferences. Also consistent with recent previous research (Bergman et al., 2017; Ellis et al., 2017), we did not find measurement instruments suitable for mapping individuals’ preferences for support. In order to be successful in helping the individual and thereby prevent ill health, more specified interventions and instruments are required that capture the individual preferences for support.

### 5.1 Methodological considerations and limitations

By integrating the studies, looking at treatments of the same phenomenon in qualitative, quantitative and mixed‐methods studies, we have created a comprehensive overview of the evidence base regarding children and adolescents’ preferences for support when living with a dying parent (Whittemore & Knafl, 2005). Although combining findings based on quantitative, qualitative and mixed‐methods is noted to be challenging, calling for a transparent process, the comprehensive understanding enabled by such an integrative review exceeds the weaknesses of the method.

In this study, we have striven to ensure validity and reliability throughout the research process by working closely together as authors. For example, pairs of authors (EB & CO or EB & ML) independently selected studies for inclusion, quality assessment and data extraction, and uncertainties and disagreements were discussed until all three authors reached consensus. Although the methodological quality of the articles varied (Nordström et al., 2016a, 2016b), some articles were included in this study in accordance with the integrative approach (Whittemore & Knafl, 2005) and the PRISMA statement (Moher et al., 2009). Our review consists of relatively up‐to‐date knowledge, as most of the included studies were conducted during the last decade. However, some older studies that have been included do not seem to differ significantly in their findings regarding children and adolescents’ preferences for support. The included studies were conducted in similar contexts, which constitutes both a strength and a weakness: the strength is that the studies are comparable; the weakness is that they do not provide knowledge of many contexts outside the Nordic countries and North America.

Since we found so few relevant studies, we also included three articles with participants over 18 years old. However, these studies also included children and adolescents in the 2–18‐year age group or were retrospective, with the participants having been children and adolescents below 19 years old when they lived with their dying parent.

## 6 CONCLUSION

The results of this study show that the children and adolescents strived for control and balance regarding involvement in the sick parent's care and treatment, as well as time spent in the home when living with the dying parent. Children and adolescents preferred emotional support from their families, especially from parents, and from friends they wanted opportunities to socialize, to do things together with other children and adolescents. A consistent perception among children and adolescents was that support from HCPs was mainly for the sick parent. However, they wanted HCP to show compassion and adjusted information on their own terms. Therefore, challenges for HCP are to identify vulnerable children and adolescents with a weak social network and provide timely individually adapted support.

Although there was a limited selection of published studies, half of the included studies had been conducted in the last 10 years, which indicates increasing activity and interest in this research field. Most of the studies concerned families where the sick parent was diagnosed with cancer, so it is unclear whether other diagnoses would have generated different children and adolescents’ preferences. The same applies to urban versus rural settings, specified in only two studies, although our findings indicate that a person‐ or child‐centred approach to meeting children and adolescents’ needs is required regardless of context.

Furthermore, there is a need to develop supportive interventions based on children and adolescents’ preferences conducted with a child‐centred perspective which acknowledge that they still are dependent on their families. In addition, there is a need to develop instruments to identify and assess children and adolescents’ preferences for support.

## CONFLICT OF INTEREST

No conflicts of interest to report.

## AUTHOR CONTRIBUTIONS

Data collection (literature search): EB; selection of literature: EB, ML and CO; quality appraisal and data extraction: EB, ML and CO; data analysis: EB, ML and CO; manuscript preparation: EB; supervision and critical review: ML and CO. All authors approved the final manuscript.

All authors have agreed on the final version and meet at least one of the following criteria [recommended by the ICMJE (http://www.icmje.org/recommendations/)]:

- substantial contributions to conception and design, acquisition of data or analysis and interpretation of data;
- drafting the article or revising it critically for important intellectual content.

## ETHICAL APPROVAL

Not applicable.

## Data Availability

Data sharing not applicable.

## References

[nop21187-bib-0001] Alvariza, A. , Lövgren, M. , Bylund‐Grenklo, T. , Hakola, P. , Fürst, C. J. , & Kreicbergs, U. (2017). How to support teenagers who are losing a parent to cancer: Bereaved young adults' advice to healthcare professionals—A nationwide survey. Palliative and Supportive Care, 15(3), 313–319. 10.1017/S1478951516000730 27692012

[nop21187-bib-0002] Bartfai Jansson, K. , & Anderzen‐Carlsson, A. (2017). Adolescents' perspectives of living with a parent's cancer: A unique and personal experience. Cancer Nursing, 40(2), 94–101. 10.1097/ncc.0000000000000358 26925993

[nop21187-bib-0003] Beale, E. A. , Sivesind, D. , & Bruera, E. (2004). Parents dying of cancer and their children. Palliative and Supportive Care, 2(4), 387–393. 10.1017/S1478951504040519 16594401

[nop21187-bib-0004] Benzein, E. G. , Hagberg, M. , & Saveman, B. I. (2008). ‘Being appropriately unusual’: A challenge for nurses in health‐promoting conversations with families. Nursing Inquiry, 15(2), 106–115. 10.1111/j.1440-1800.2008.00401.x 18476853

[nop21187-bib-0005] Bergersen, E. , Larsson, M. , & Olsson, C. (2020). Living with a dying parent. Children and young people’s preferences for support: an integrative review. PROSPERO 2020 CRD42020130943. https://www.crd.york.ac.uk/PROSPERO/display_record.php?RecordID=130943 10.1002/nop2.1187 PMC8994933 35156340

[nop21187-bib-0006] Bergman, A.‐S. , Axberg, U. , & Hanson, E. (2017). When a parent dies: A systematic review of the effects of support programs for parentally bereaved children and their caregivers. BMC Palliative Care, 16(39), 1–15. 10.1186/s12904-017-0223-y 28797262 PMC5553589

[nop21187-bib-0007] Berman, H. , Cragg, C. E. , & Kuenzig, L. (1988). Having a parent die of cancer: Adolescents' grief reactions. Oncology Nursing Forum, 15(2), 159–163. 3357828

[nop21187-bib-0008] Brent, D. A. , Melhem, N. M. , Masten, A. S. , Porta, G. , & Payne, M. W. (2012). Longitudinal effects of parental bereavement on adolescent developmental competence. Journal of Clinical Child and Adolescent Psychology, 41(6), 778–791. 10.1080/15374416.2012.717871 23009724 PMC3493857

[nop21187-bib-0009] Buchwald, D. , Delmar, C. , & Schantz‐Laursen, B. (2012). How children handle life when their mother or father is seriously ill and dying. Scandinavian Journal of Caring Sciences, 26(2), 228–235. 10.1111/j.1471-6712.2011.00922.x 21950563

[nop21187-bib-0010] Bugge, K. E. , Helseth, S. , & Darbyshire, P. (2008). Children's experiences of participation in a family support program when their parent has incurable cancer. Cancer Nursing, 31(6), 426–434. 10.1097/01.NCC.0000339250.83571.b0 18987509

[nop21187-bib-0011] Bylund‐Grenklo, T. , Kreicbergs, U. , Uggla, C. , Valdimarsdóttir, U. A. , Nyberg, T. , Steineck, G. , & Fürst, C. J. (2015). Teenagers want to be told when a parent's death is near: A nationwide study of cancer‐bereaved youths’ opinions and experiences. Acta Oncologica, 54(6), 944–950. 10.3109/0284186X.2014.97889 25467964

[nop21187-bib-0012] Chen, C.‐Y.‐C. , & Panebianco, A. (2018). Interventions for young bereaved children: A systematic review and implications for school mental health providers. Child & Youth Care Forum, 47(2), 151–171. 10.1007/s10566-017-9426-x

[nop21187-bib-0013] Christ, G. H. , Siegel, K. , & Sperber, D. (1994). Impact of parental terminal cancer on adolescents. American Journal of Orthopsychiatry, 64(4), 604–613. 10.1037/h0079569 7847576

[nop21187-bib-0014] Coyne, I. , Holmström, I. , & Söderbäck, M. (2018). Centeredness in healthcare: A concept synthesis of family‐centered care, person‐centered care and child‐centered care. Journal of Pediatric Nursing, 42, 45–56. 10.1016/j.pedn.2018.07.001 30219299

[nop21187-bib-0015] Dehlin, L. , & Reg, L. M. (2009). Adolescents' experiences of a parent's serious illness and death. Palliative and Supportive Care, 7(1), 13–25. 10.1017/S1478951509000042 19619371

[nop21187-bib-0016] Dumont, I. , & Kissane, D. (2009). Techniques for framing questions in conducting family meetings in palliative care. Palliative and Supportive Care, 7(2), 163–170. 10.1017/S1478951509000212 19538798

[nop21187-bib-0017] EAPC . (2009). White Paper on standards and norms for hospice and palliative care in Europe: Part 1. Recommendations from the European Association for Palliative Care. European Journal of Palliative Care, 17, 22–33.

[nop21187-bib-0018] Eklund, R. , Jalmsell, L. , Kreicbergs, U. , Alvariza, A. , & Lövgren, M. (2020). Children’s experiences of the family talk intervention when a parent is cared for in palliative home care—A feasibility study. Death Studies, 1–12. 10.1080/07481187.2020.1829747 33054633

[nop21187-bib-0019] Eklund, R. , Kreicbergs, U. , Alvariza, A. , & Lövgren, M. (2020). Children’s self‐reports about illness‐related information and family communication when a parent has a life‐threatening illness. Journal of Family Nursing, 26(2), 102–110. 10.1177/1074840719898192 31931660

[nop21187-bib-0020] Ellis, S. , Wakefield, C. , Antill, G. , Burns, M. , & Patterson, P. (2017). Supporting children facing a parent's cancer diagnosis: A systematic review of children's psychosocial needs and existing interventions. European Journal of Cancer Care, 26(1), e12432. 10.1111/ecc.12432 26776913

[nop21187-bib-0021] Elmberger, E. , Bolund, C. , Magnusson, A. , Lützén, K. , & Andershed, B. (2008). Being a mother with cancer: Achieving a sense of balance in the transition process. Cancer Nursing, 31(1), 58–66. 10.1097/01.NCC.0000305677.90963.67 18176133

[nop21187-bib-0022] Etkind, S. N. , Bone, A. E. , Gomes, B. , Lovell, N. , Evans, C. J. , Higginson, I. J. , & Murtagh, F. E. M. (2017). How many people will need palliative care in 2040? Past trends, future projections and implications for services. BMC Medicine, 15(1), 102. 10.1186/s12916-017-0860-2 28514961 PMC5436458

[nop21187-bib-0023] Golsäter, M. , Enskär, K. , & Knutsson, S. (2019). Parents’ perceptions of how nurses care for children as relatives of an ill patient: Experiences from an oncological outpatient department. European Journal of Oncology Nursing, 39, 35–40. 10.1016/j.ejon.2019.01.004 30850136

[nop21187-bib-0024] Gupta, T. (2018). Psychological management of bereavement among adolescents: A case series. Journal of Indian Association for Child & Adolescent Mental Health, 14(2), 117–127.

[nop21187-bib-0025] Higgins, J. P. , & Green, S. (2008). Cochrane handbook for systematic reviews of interventions. UK Wiley‐Blackwell.

[nop21187-bib-0026] Høeg, B. L. , Johansen, C. , Christensen, J. , Frederiksen, K. , Dalton, S. O. , Bøge, P. , Dencker, A. , Dyregrov, A. , & Bidstrup, P. E. (2019). Does losing a parent early influence the education you obtain? A nationwide cohort study in Denmark. Journal of Public Health, 41(2), 296–304. 10.1093/pubmed/fdy070 29684221

[nop21187-bib-0027] Hudson, P. , Quinn, K. , O'Hanlon, B. , & Aranda, S. (2008). Family meetings in palliative care: Multidisciplinary clinical practice guidelines. BMC Palliative Care, 7(1), 12. 10.1186/1472-684X-7-12 18710576 PMC2542352

[nop21187-bib-0028] Jakobsen, I. S. , & Christiansen, E. (2011). Young people’s risk of suicide attempts in relation to parental death: A population‐based register study. Journal of Child Psychology and Psychiatry, 52(2), 176–183. 10.1111/j.1469-7610.2010.02298.x 21039482

[nop21187-bib-0029] Jansson, K. B. , & Anderzén‐Carlsson, A. (2017). Adolescents’ perspectives of living with a Parent’s cancer: A unique and personal experience. Cancer Nursing, 40(2), 94–101. 10.1097/ncc.0000000000000358 26925993

[nop21187-bib-0030] Karlsson, E. , Andersson, K. , & Ahlstrom, B. H. (2013). Loneliness despite the presence of others – Adolescents' experiences of having a parent who becomes ill with cancer. European Journal of Oncology Nursing, 17(6), 697–703. 10.1016/j.ejon.2013.09.005 24183584

[nop21187-bib-0031] Keeley, M. , & Baldwin, P. (2012). Final conversations, phase 2: Children and everyday communication. Journal of Loss and Trauma, 17(4), 376–387. 10.1080/15325024.2011.650127

[nop21187-bib-0032] Kopchak Sheehan, D. , Burke Draucker, C. , Christ, G. H. , Murray Mayo, M. , Heim, K. , & Parish, S. (2014). Telling adolescents a parent is dying. Journal of Palliative Medicine, 17(5), 512–520. 10.1089/jpm.2013.0344 24745829 PMC4012636

[nop21187-bib-0033] Kralik, D. , Visentin, K. , & Van Loon, A. (2006). Transition: A literature review. Journal of Advanced Nursing, 55(3), 320–329. 10.1111/j.1365-2648.2006.03899.x 16866826

[nop21187-bib-0034] Kühne, F. , Krattenmacher, T. , Beierlein, V. , Grimm, J. C. , Bergelt, C. , Romer, G. , & Möller, B. (2012). Minor children of palliative patients: A systematic review of psychosocial family interventions. Journal of Palliative Medicine, 15(8), 931–945. 10.1089/jpm.2011.0380 22849598 PMC3396138

[nop21187-bib-0035] Marcussen, J. , Thuen, F. , O'Connor, M. , Wilson, R. L. , & Hounsgaard, L. (2020). Double bereavement, mental health consequences and support needs of children and young adults—When a divorced parent dies. Journal of Clinical Nursing, 29(7–8), 1238–1253. 10.1111/jocn.15181 31910291

[nop21187-bib-0036] McFarlane, J. , & Liu, F. (2020). The lived experiences of family caregivers of persons dying in home hospice: Support, advocacy, and information urgently needed. Journal of Hospice and Palliative Nursing, 22(2), 145–151. 10.1097/NJH.0000000000000632 32011356

[nop21187-bib-0037] Melcher, U. , Sandell, R. , & Henriksson, A. (2015). Maintaining everyday life in a family with a dying parent: Teenagers’ experiences of adapting to responsibility. Palliative and Supportive Care, 13(6), 1595–1601. 10.1017/S1478951515000085 25800062

[nop21187-bib-0038] Meleis, A. I. (2010). Transitions theory: Middle range and situation specific theories in nursing research and practice. Springer Pub.

[nop21187-bib-0039] Meleis, A. I. , Sawyer, L. M. , Im, E.‐O. , Messias, D. K. H. , & Schumacher, K. (2000). Experiencing transitions: An emerging middle‐range theory. Advances in Nursing Science, 23(1), 12–28. 10.1097/00012272-200009000-00006 10970036

[nop21187-bib-0040] Moher, D. , Liberati, A. , Tetzlaff, J. , & Altman, D. G. (2009). Preferred reporting items for systematic reviews and meta‐analyses: The PRISMA statement. Annals of Internal Medicine, 151(4), 264–269. 10.7326/0003-4819-151-4-200908180-00135 19622511

[nop21187-bib-0041] Nickerson, A. , Bryant, R. A. , Aderka, I. M. , Hinton, D. E. , & Hofmann, S. G. (2013). The impacts of parental loss and adverse parenting on mental health: Findings from the National Comorbidity Survey‐Replication. Psychological Trauma: Theory, Research, Practice, and Policy, 5(2), 119–127. 10.1037/a0025695

[nop21187-bib-0042] Niemelä, M. , Väisänen, L. , Marshall, C. , Hakko, H. , & Räsänen, S. (2010). The experiences of mental health professionals using structured family‐centered interventions to support children of cancer patients. Cancer Nursing, 33(6), E18–E27. 10.1097/NCC.0b013e3181ddfcb5 20555258

[nop21187-bib-0043] Nilsson, M. E. , Maciejewski, P. K. , Zhang, B. , Wright, A. A. , Trice, E. D. , Muriel, A. C. , Friedlander, R. J. , Fasciano, K. M. , Block, S. D. , & Prigerson, H. G. (2009). Mental health, treatment preferences, advance care planning, location, and quality of death in advanced cancer patients with dependent children. Cancer, 115(2), 399–409. 10.1002/cncr.24002 19110677 PMC2630701

[nop21187-bib-0044] Nordström, G. , Wilde‐Larsson, B. , Sandsdalen, T. , & Jansson, J. (2016a). Assessment tool‐ qualitative studies. Department of Health Science, Faculty of Health, Science and Technology, Karlstad University (in Swedish). Unpublished.

[nop21187-bib-0045] Nordström, G. , Wilde‐Larsson, B. , Sandsdalen, T. , & Jansson, J. (2016b). Assessment tool – quantitative studies (not RCT). Department of Health Science, Faculty of Health, Science and Technology, Karlstad University (in Swedish). Unpublished.

[nop21187-bib-0046] Northouse, L. L. , Katapodi, M. C. , Song, L. , Zhang, L. , & Mood, D. W. (2010). Interventions with family caregivers of cancer patients: Meta‐analysis of randomized trials. CA: A Cancer Journal for Clinicians, 60(5), 317–339. 10.3322/caac.20081 20709946 PMC2946584

[nop21187-bib-0047] Office of the High Commissioner for Human Rights . (1989). Convention on the rights of the child. https://www.ohchr.org/Documents/ProfessionalInterest/crc.pdf

[nop21187-bib-0048] Ouzzani, M. , Hammady, H. , Fedorowicz, Z. , & Elmagarmid, A. (2016). Rayyan—A web and mobile app for systematic reviews. Systematic Reviews, 5(1), 210. 10.1186/s13643-016-0384-4 27919275 PMC5139140

[nop21187-bib-0049] Patterson, P. , & Rangganadhan, A. (2010). Losing a parent to cancer: A preliminary investigation into the needs of adolescents and young adults. Palliat Support Care, 8(3), 255–265. 10.1017/s1478951510000052 20875169

[nop21187-bib-0050] Phillips, F. (2014). Adolescents living with a parent with advanced cancer: A review of the literature. Psycho‐Oncology, 23(12), 1323–1339. 10.1002/pon.3570 24911540

[nop21187-bib-0051] Phillips, F. , & Lewis, F. M. (2015). The adolescents experience when a parent has advanced cancer: A qualitative inquiry. Palliative Medicine, 29(9), 851–858. 10.1177/0269216315578989 25855631

[nop21187-bib-0052] Popplestone‐Helm, S. V. , & Helm, D. P. (2009). Setting up a support group for children and their well carers who have a significant adult with a life‐threatening illness. International Journal of Palliative Nursing, 15(5), 214–221. 10.12968/ijpn.2009.15.5.42346 19491746

[nop21187-bib-0053] Punziano, A. C. , Piredda, M. , Mastroianni, C. , Fiorelli, F. R. , & De Marinis, M. G. (2017). Health professional's experiences of supporting teenagers who have lost a parent. Journal of Hospice and Palliative Nursing, 19(5), 415–423. 10.1097/NJH.0000000000000360

[nop21187-bib-0054] Rosner, R. , Kruse, J. , & Hagl, M. (2010). A meta‐analysis of interventions for bereaved children and adolescents. Death Studies, 34(2), 99–136. 10.1080/07481180903492422 24479177

[nop21187-bib-0055] Rostila, M. , Berg, L. , Arat, A. , Vinnerljung, B. , & Hjern, A. (2016). Parental death in childhood and self‐inflicted injuries in young adults‐a national cohort study from Sweden. European Child and Adolescent Psychiatry, 25(10), 1103–1111. 10.1007/s00787-016-0833-6 26932156

[nop21187-bib-0056] Salam, R. A. , Das, J. K. , Lassi, Z. S. , & Bhutta, Z. A. (2016). Adolescent health interventions: Conclusions, evidence gaps, and research priorities. Journal of Adolescent Health, 59(4), S88–S92. 10.1016/j.jadohealth.2016.05.006 PMC5026678 27664599

[nop21187-bib-0057] Saldinger, A. , Cain, A. , & Porterfield, K. (2003). Managing traumatic stress in children anticipating parental death. Psychiatry: Interpersonal and Biological Processes, 66(2), 168–181. 10.1521/psyc.66.2.168.20613 12868295

[nop21187-bib-0058] Sawyer, S. M. , Afifi, R. A. , Bearinger, L. H. , Blakemore, S.‐J. , Dick, B. , Ezeh, A. C. , & Patton, G. C. (2012). Adolescence: A foundation for future health. The Lancet, 379(9826), 1630–1640. 10.1016/S0140-6736(12)60072-5 22538178

[nop21187-bib-0059] Semmens, J. , & Peric, J. (1995). Children's experience of a parent's chronic illness and death. Australian Journal of Advanced Nursing, 13(2), 30–38. 8694999

[nop21187-bib-0060] Shallcross, A. J. , Visvanathan, P. D. , McCauley, R. , Clay, A. , & van Dernoot, P. R. (2016). The effects of the CLIMB(R) program on psychobehavioral functioning and emotion regulation in children with a parent or caregiver with cancer: A pilot study. Journal of Psychosocial Oncology, 34(4), 259–273. 10.1080/07347332.2016.1191577 27355243

[nop21187-bib-0061] Sheehan, D. K. , Mayo, M. M. , Christ, G. H. , Heim, K. , Parish, S. , Shahrour, G. , & Draucker, C. B. (2016). Two worlds: Adolescents' strategies for managing life with a parent in hospice. Palliative and Supportive Care, 14(3), 177–186. 10.1017/S1478951515000735 26126748 PMC12096904

[nop21187-bib-0062] Simons, R. L. , Woodring, D. , Simons, L. G. , Sutton, T. E. , Lei, M.‐K. , Beach, S. R. H. , Barr, A. B. , & Gibbons, F. X. (2019). Youth adversities amplify the association between adult stressors and chronic inflammation in a domain specific manner: Nuancing the early life sensitivity model. Journal of Youth and Adolescence, 48(1), 1–16. 10.1007/s10964-018-0977-4 30603835 PMC7685217

[nop21187-bib-0063] Spuij, M. , Dekovic, M. , & Boelen, P. A. (2015). An open trial of 'Grief‐Help': A cognitive‐behavioural treatment for prolonged grief in children and adolescents. Clinical Psychology and Psychotherapy, 22(2), 185–192. 10.1002/cpp.1877 24227661

[nop21187-bib-0064] Sveen, J. , Kreicbergs, U. , Melcher, U. , & Alvariza, A. (2016). Teenagers' reasoning about a parent's recent death in cancer. Palliative and Supportive Care, 14(4), 349–357. 10.1017/S1478951515001054 26462758

[nop21187-bib-0065] Tillquist, M. , Backrud, F. , & Rosengren, K. (2016). Dare to ask children as relatives! A qualitative study about female teenagers' experiences of losing a parent to cancer. Home Health Care Management & Practice, 28(2), 94–100. 10.1177/1084822315610104

[nop21187-bib-0066] Torbic, H. (2011). Children and grief: But what about the children? Home Healthcare Nurse, 29(2), 67–77. 10.1097/NHH.0b013e31820861dd 21301271

[nop21187-bib-0067] Valen, K. , Haug, B. , Holm, A. L. , Toverud Jensen, K. , & Grov, E. K. (2020). From palliative care developed during simulation, to performance in clinical practice‐descriptions from nursing students. Journal of Hospice and Palliative Nursing, 22(3), 204–212. 10.1097/NJH.0000000000000644 32282556

[nop21187-bib-0068] Warnick, A. L. (2015). Supporting youth grieving the dying or death of a sibling or parent: Considerations for parents, professionals, and communities. Current Opinion in Supportive & Palliative Care, 9(1), 58–63. 10.1097/spc.0000000000000115 25581448

[nop21187-bib-0069] Werner‐Lin, A. , Biank, N. M. , & Rubenstein, B. (2010). There’s no place like home: Preparing children for geographical and relational attachment disruptions following parental death to cancer. Clinical Social Work Journal, 38(1), 132–143. 10.1007/s10615-009-0233-1

[nop21187-bib-0070] Whittemore, R. , & Knafl, K. (2005). The integrative review: Updated methodology. Journal of Advanced Nursing, 52(5), 546–553. 10.1111/j.1365-2648.2005.03621.x 16268861

[nop21187-bib-0071] World Health Organization . (2001). The second decade: Improving adolescent health and development. https://apps.who.int/iris/bitstream/handle/10665/64320/WHO_FRH_ADH_98.18_Rev.1pdf;jsessionid=C9975B0CEA4409B2AEF34176B1DBCDC1?sequence=1

